# Development of a Multiplex Autoantibody Test for Detection of Lung Cancer

**DOI:** 10.1371/journal.pone.0095444

**Published:** 2014-04-22

**Authors:** Jing Jia, Wenzhe Wang, Wen Meng, Mingjian Ding, Shenglin Ma, Xiaoju Wang

**Affiliations:** 1 Center for Molecular Medicine, Zhejiang Academy of Medical Sciences, Hangzhou, Zhejiang, P. R. China; 2 Institute of Lung Cancer, Zhejiang Academy of Medical Sciences, Hangzhou, Zhejiang, P. R. China; 3 Hangzhou First People’s Hospital, Hangzhou, Zhejiang, P. R. China; University of Pittsburgh, United States of America

## Abstract

Lung cancer is the leading cause of cancer-related deaths for both men and women. Early diagnosis of lung cancer has a 5-year survival rate of 48.8%, however, nearly 35% of stage I patients relapses after surgical resection, thus portending a poor prognosis. Therefore, detecting lung cancer in early stage and further identifying the high-risk patients would allow the opportunity to provide adjuvant therapy and possibly increase survival. There is considerable evidence that the immune system produces an autoantibody response to neoplastic cells. The detection of such autoantibodies has been shown to have diagnostic and prognostic value. Here we took advantage of the high-throughput Luminex technique to multiplex a total of 14 tumor-associated autoantigens to detect the autoantibody from the patients sera. The 14 antigens were expressed by in vitro transcription/translation system with HaloTag at N-terminus. The fusion proteins were then covalently immobilized onto the Luminex microspheres conjugated by the halo-link ligand, thus eliminating the protein purification procedure. Sera samples from cancer patients and healthy controls were interacted with the microsphere-antigen complex to measure the autoantibodies. We have developed a quick multiplex detection system for measuring autoantibody signature from patient sera with minimal cross-reaction. A panel of seven autoantibody biomarkers has generated an AUC>80% in distinguishing the lung cancers from healthy controls. This study is the first report by combining Luminex platform and HaloTag technology to detect humoral immune response in cancer patients. Due to the flexibility of the Luminex technology, this approach can be applied to others conditions such as infectious, neurological, and metabolic diseases. One can envision that this multiplex Luminex system as well as the panel of seven biomarkers could be used to screen the high-risk population with subsequent CT test based on the blood test result.

## Introduction

Lung cancer is the leading cause of cancer-related deaths for both men and women worldwide. In 2012, it was estimated that 226,160 people would be diagnosed with lung cancer and 160,340 patients would die from their disease. The current 5-year overall survival rate for patients is 16% until 2007, among the lowest of all cancers, which has improved only marginally in the past decade [1]. Most lung cancer patients are diagnosed at the late/distant stages thus with low survival rate, due to the lack of perceivable symptoms at the early stage of tumorigenesis [2].

The data suggest that diagnosing lung cancers when it is small and locally defined may increase the cure chance [3], [4]. Early diagnosis of lung cancer could significantly improve the 5-year survival rate up to 48.8% compared to 3.3% of late/distant stage [5]. Therefore, detection of early-stage lung cancer and the identification of high-risk patients would provide potential adjuvant therapy and possibly increase survival.

The current methods for the diagnosis of lung cancer require a biopsy and pathologic examination of the tissue usually after discovery of the lesion on chest X-ray or computerized tomography. There is currently no blood test available for lung cancer. Several approaches have been developed to identify biomarkers for diagnosis of lung cancer [6], [7]. Among these, the immune system have been shown to produce autoantibodies against neoplastic cells as well as dysregulated tumor associated antigens [8], [9], therefore detection of such autoantibodies have diagnostic and prognostic value [10], [11]. Most importantly, humoral immune responses exist several months or years prior to the clinical symptoms, thus the autoantibodies could be used for early diagnosis of cancer patients [7], [9], [12]. Additionally, it is reported that B cells and B cell-associated antibodies promote de novo carcinogenesis [13], suggesting that the humoral immune response may play a direct role in cancer progression.

**Figure 1:**
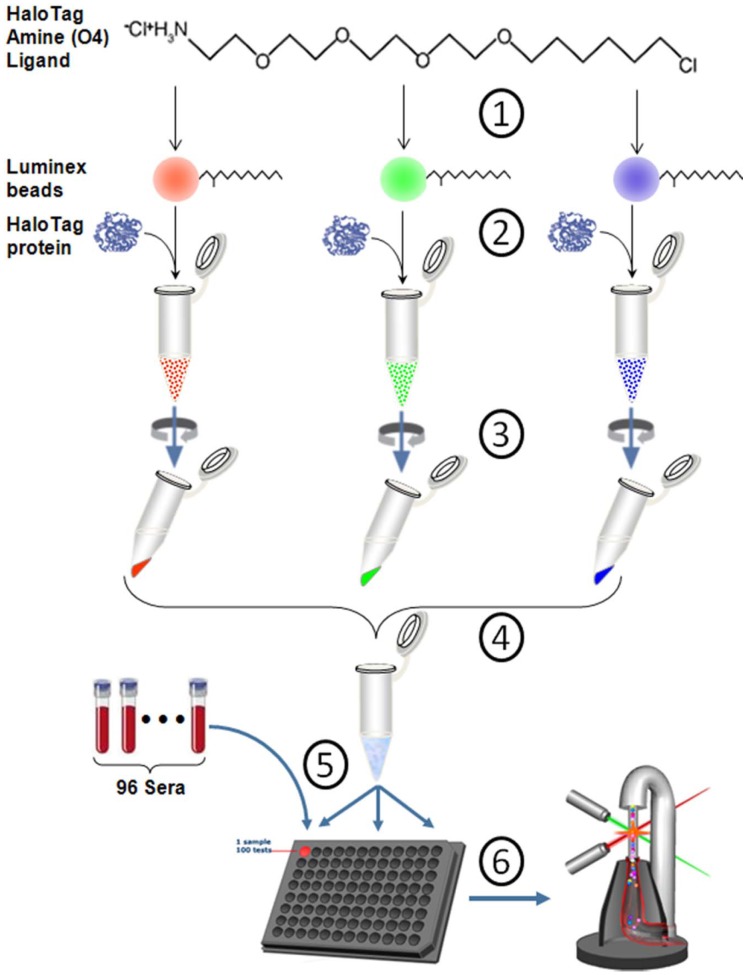
The flowchart of multiplex autoantibody by the Luminex xMap system. (1) The HaloTag Amine Ligands were conjugated to Luminex magnetic beads via the activated carboxylic acid of COOH radical group. Different color represents various types of Luminex beads. (2) The recombinant Halo-tagged proteins were covalently immobilized onto the Luminex beads conjugated with HaloTag ligand via reactive chloroalkane linkers on ligands. Each bead was immobilized with different protein separately. (3)–(4) Due to the covalent binding, each bead-protein complex individually went through vigorously washing and then combined to make the microsphere pool. (5) The protein-coupled microsphere pool was then equally aliquotted into a 96-well plate. Each well contains multiple autoantigen biomarkers and can be used for a single serum sample. (6) The autoantibodies in sera bound to the antigen conjugated microspheres, and were detected by R-phycoerythrin conjugated anti-human antibody. The signal was read on Luminex 200 bioanalyzer.

In this study, we described a non-invasive diagnostic system on Luminex xMAP platform to detect serum autoantibody for diagnosis of lung cancer. The candidate autoantigens were selected from literature and expressed by in vitro transcription/translation system using HaloTag at N-terminus. Without purification, the recombinant proteins were covalently immobilized onto Luminex bead. Serum samples were incubated with the bead-autoantigen complex to measure the amount of autoantibodies. A panel of seven antigens, namely p53, NY-ESO-1, Livin, Ubiquilin1, BIRC, p62 and PRDX, were used in a multivariate statistical model to distinguish cancer patients from healthy controls. This is the first study of detecting multiplex autoantibody on Luminex technology.

## Materials and Methods

### Expression of Recombinant Proteins

The cDNAs for 14 autoantigens were cloned into the Flexi vector (Promega, USA) with a HaloTag at N-terminus. The Halo tagged recombinant proteins were expressed by in vitro transcription/translation system (Promega). The HaloTag protein was also produced as negative protein. The proteins were detected by Western blot using rabbit anti-HaloTag antibody (Promega).

### Western Blot Assay

Halo tagged recombinant proteins were expressed by *in vitro* transcription/translation system, and then subjected for Western Blot. Briefly, a total of 3 µl protein lysate was loaded onto 12% SDS-PAGE, then electrophoretically transferred to PVDF membrane (GE). The protein lysate with the HaloTag protein only (31 kDa) was loading as control. After blocking, the membranes were blotted with rabbit anti-HaloTag antibody (Promega), followed by incubation with horseradish peroxidase-conjugated secondary antibody (Proteintech). The protein signals were detected by ECL reagent (GE), and photographed using Alpha Innotech Fluor Chem FC2 chemiluminescence image analysis system (Alpha Innotech).

### Patient Serum Samples

The study was approved by the Institutional Review Board of Zhejiang Academy of Medicine Sciences. All participants who provided sera were given written informed consents.

The patients with localized lung cancer were collected at the Department of Oncology, the Hangzhou first people’s hospital from August 2010 to March 2012. A total of 117 patient samples met the following eligibility criteria for this study: 1) from biopsy-proven clinically localized lung cancer, 2) no prior lung cancer therapy, 3) date of serum drawn was immediately prior to date of surgery, 4) no other concurrent malignant diseases and autoimmune diseases. Of these 117 samples, 44 samples were chosen at random to be used in the development and validation of Luminex platform, while 25 samples were used for biomarker selection, and the rest 48 samples were used for multiplex biomarker analysis. Similarly, to serve as control subjects, the hospital provided 110 sera samples in the age range of 29–76 with no history of cancer collected between 2011 and 2012. These 110 samples were also randomly divided into three groups in this study, specifically 35 samples for Luminex development, 25 samples for biomarker selection and 50 samples for multiplex analysis.

### Conjugation of the HaloTag Amine Ligand to Luminex Microsphere

The minimum quantity of HaloTag Amine (O4) Ligand (Promega,USA) for the maximum conjugation efficiency on the Luminex microspheres (Luminex Corp.) was determined as following. A total of 5×10^6^ Luminex magnetic microspheres were conjugated with a series of dilution of HaloTag Amine Ligand: 0.0016 mg, 0.008 mg, 0.04 mg, 0.2 mg, 1 mg. Briefly, the microspheres were suspended in a solution of 100 mmol/L MES (pH 6.0), containing 5 mg/mL 1-ethyl-3-(3-dimethylaminopropyl) carbodiimide (Thermo Scientific) and HaloTag Amine Ligand. After a 2-hour incubation in the dark, the microspheres were washed and resuspended in 100 mmol/L MES (pH 4.5), and stored at 4°C.

The Luminex microspheres coupled with HaloTag Ligand were incubated with purified HaloTag protein in a series of dilution from 0 µg to 5 µg. The MFI (mean fluorescence intensity) represents the binding signal and was detected by anti-HaloTag antibody.

### Development and Validation of the Protein-microsphere Complex

The ligand-microsphere complex in different bead regions was separately incubated with recombinant HaloTag fusion proteins at ambient temperature in dark for 60 minute. Following incubation, the microspheres were washed with PBS containing 0.1% BSA and 0.5% Tween 20, and resuspended in PBS containing 0.1% BSA for validation.

The amount of protein conjugated to the microsphere was measured using commercial antibody (rabbit anti-p62 antibody, rabbit anti-p53 antibody, Santa Cruz) against each target protein. Briefly, serial dilutions of each antibody (ranging from 0 to 5 µg/mL in PBS, 1% BSA) were incubated with 3,000 microspheres conjugated with different proteins in a 96-well plate for 1 hour. Following washing with PBS/1% BSA, the microspheres were incubated with R-phycoerythrin conjugated secondary antibody for 30 minutes. The resulting complex was again washed and resuspended in PBS/1% BSA before reading on Luminex 200 bioanalyzer (Luminex Corp). The MFI value represents the binding signal.

To test the detection ability of the Luminex system, 44 cancer and 35 health control sera samples were randomly selected, and the NY-ESO-1 autoantibody was measured by Luminexe system. Briefly, different microspheres conjugated with recombinant NY-ESO-1 and HaloTag separately were combined and aliquoted into a 96-well plate. The serum samples were diluted at 1∶200 in PBS/1% BSA and incubated with the beads overnight at 4C with shaking. The autoantibody signals were then detected by R-phycoerythrin conjugated anti-human polyclonal antibody (Rockland) for 0.5 hour with constant agitation. After three washes, the reaction complex was resuspended in PBS/1% BSA for reading on Luminex 200 bioanalyzer. The MFI values were represented by MFI(NY-ESO-1) minus MFI(HaloTag).

The fluorescence intensities were also compared with a commercial ELISA kit (Biovalue, USA). The experiment was carried out according to the manufacturer instruction.

### Detection of Autoantibody in Serum

Different microspheres conjugated with recombinant proteins and HaloTag separately were combined and aliquoted into a 96-well plate to detect the autoantibody in serum. The serum samples were diluted at 1∶200 in PBS/1% BSA and incubated with the beads overnight at 4°C with shaking. The autoantibody signals were then detected by Luminex as previously described.

### Statistical Analysis

Both univariate and multivariate analyses were used. Differences between two groups were evaluated by Student’s t-test. The correlation between single-plex and multi-plex systems or between ELISA and Luminex single-plex system were evaluated using Pearson’s correlation coefficient (R). Class prediction was examined using the BRB-Array Tools package (version 3.6) available at http://linus.nci.nih.gov/BRB-ArrayTools.html. All computations were performed using R statistical programming language (http://cran.r-project.org/) and the Bioconductor packages. Statistical analysis was performed using the software SPSS 13.0(SPSS), GraphPad Prism 5.0, and Excel. p<0.05 was considered as statistically significant.

## Results

### The General Approach of the Study is Depicted in Fig. 1

**Figure 2:**
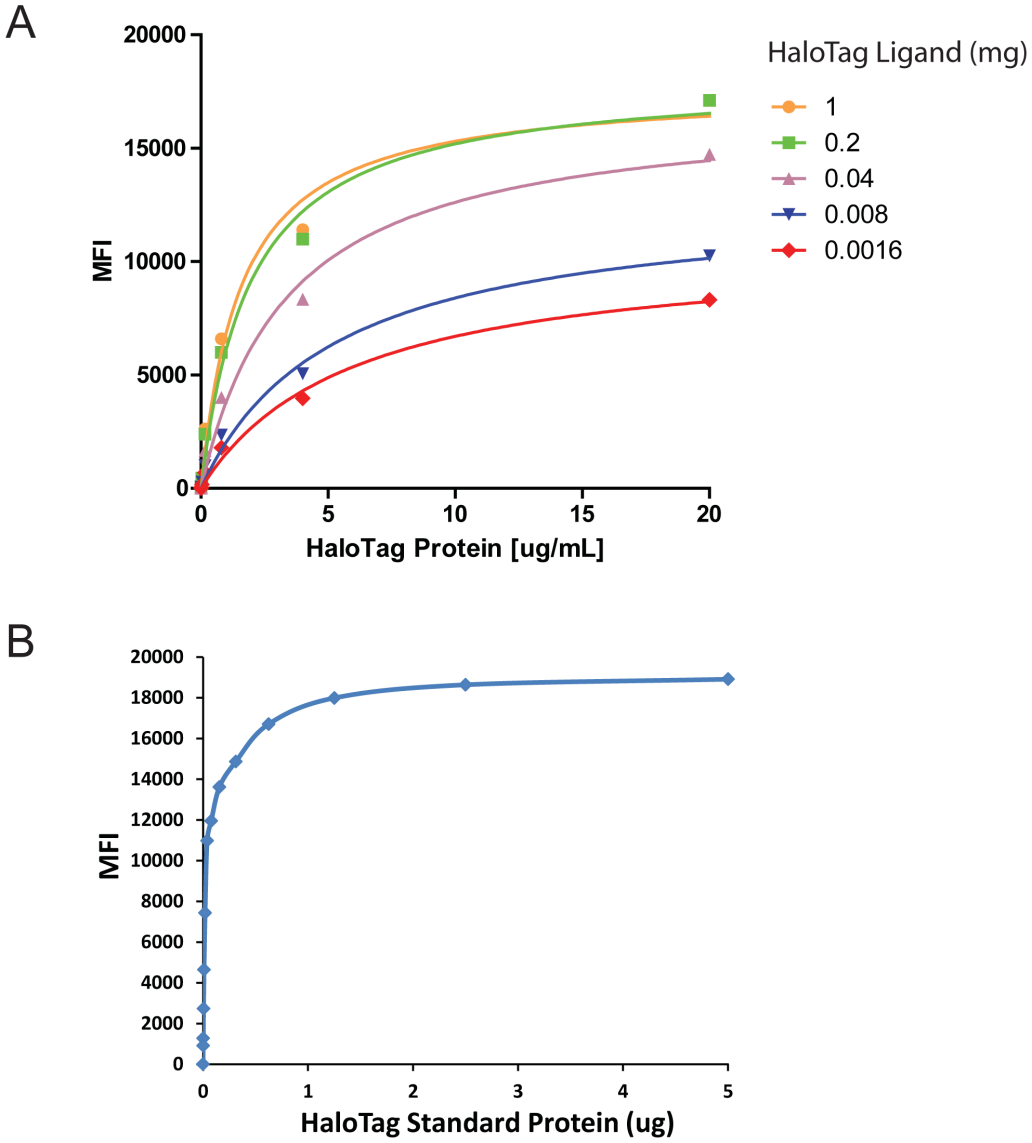
Conjugation and validation of the HaloTag Amine Ligand. (A) Titration of the HaloTag Amine Ligand conjugated on the Luminex microspheres. The purified HaloTag standard protein was used to detect the efficiency of conjugation in a series of concentrations. The MFI (mean fluorescence intensity) represents the binding signal detected by anti-HaloTag antibody. (B) Validation of the Luminex microsphere complex coupled with HaloTag Amine Ligand. The Luminex beads were coupled with 0.2 mg of the HaloTag Ligand and the signals were detected as (A).

In order to covalently immobilize the recombinant proteins to the Lumimex microsphere, the HaloTag amine ligand was first conjugated to the Luminex microspheres via the activated carboxylic acid of COOH radical group at different concentration (Fig. 2A). The coupling efficiency increased in a concentration dependent manner as detected by HaloTag standard protein (Fig. 2A). In addition, the MFI reached to the plateau at 0.2 mg and 1 mg of amine ligand, indicating the saturation of the amine ligand on the bead surface. In this study, 0.2 mg of ligand was used to conjugate the microsphere.

The Luminex microsphere conjugated by 0.2 mg of amine ligand was further confirmed using HaloTag standard protein at different concentration. The data demonstrated that the maximum MFI even at 1.25 µg of the standard protein demonstrating the optimal conjugation condition (**Fig. 2B**).

**Figure 3:**
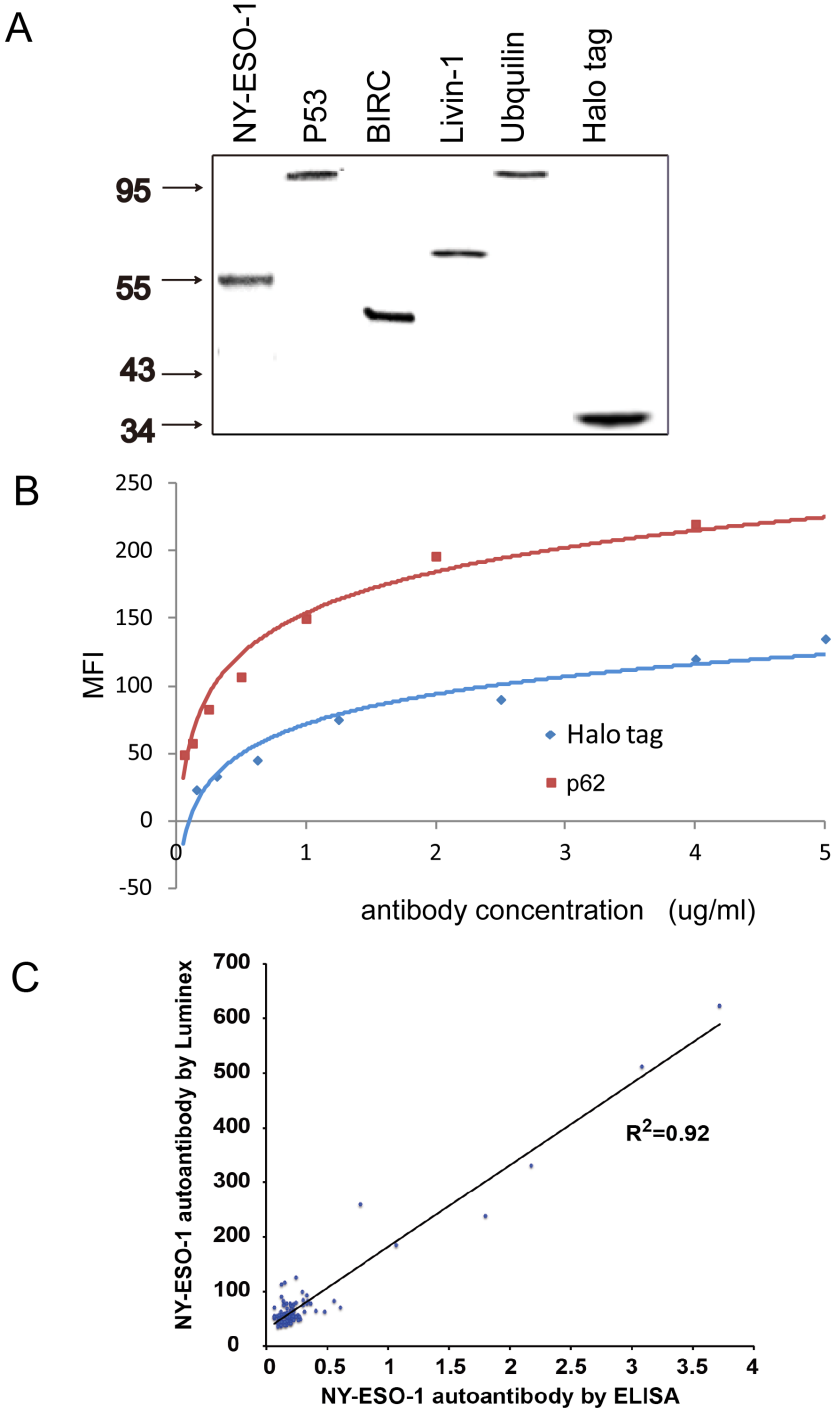
Comparison of Luminex and ELISA technologies. (A) The representative Western Blot of the recombinant autoantigens with HaloTag at N-terminus expressed by in vitro transcription/translation system. (B) Validation of the Luminex microspheres coupled with the recombinant HaloTag proteins. The immobilized proteins were detected by anti-p62 and anti-HaloTag antibodies separately and the signals were in a concentration dependent fashion. (C) Comparison of the NY-ESO-1 autoantibody in sera detected by Luminex and ELISA. A total of 44 lung cancers and 35 healthy controls were randomly selected to compare the NY-ESO-1 autoantibody measured by Luminex system and ELISA assay. R^2^ = 0.92 indicates the high correlation between Luminex and ELISA platforms in detecting NY-ESO-1 autoantibody.

To quickly screen the candidate autoantigens, a panel of 14 proteins was expressed by *in vitro* transcription/translation system. After confirmed by Western blot (**Fig. 3A**), the recombinant fusion proteins with the HaloTag were directly incubated with the microsphere-ligand complex without protein purification. The covalent bond between HaloTag and ligand forms rapidly under general physiological conditions and is highly specific and essentially irreversible, yielding a complex that is stable even under stringent conditions. The protein amount immobilized to the individual Luminex microsphere was then examined using the commercial antibodies against the corresponding antigen proteins. As shown in **Fig. 3B**, the MFI signals were measured in a concentration dependent fashion detected by anti-p62 and anti-HaloTag antibodies.

To investigate whether the Luminex system can detect the autoantibody from human specimen, 44 lung cancer and 35 healthy serum samples were randomly selected and the NY-ESO-1 autoantibody was compared using the Luminex system and a commercial ELISA kit (Biovalue, USA). Both assay platforms detected the significant higher amount of the NY-ESO-1 autoantibody in cancer patients compared to healthy controls (**Fig. 3C**). Unary linear regression analysis revealed that the correlation coefficient (Pearson r) was 0.92 for NY-ESO-1 antibodies measured by both Luminex and ELISA techniques, suggesting that two methods had a high degree of agreement in detecting NY-ESO-1 autoantibody (*p*<0.01).

**Figure 4:**
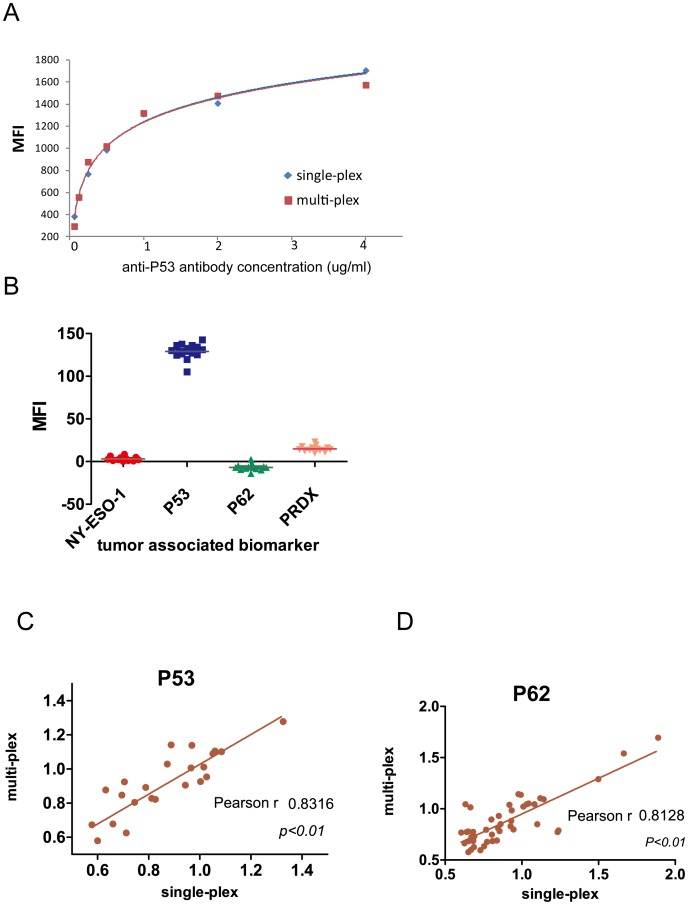
No cross-reaction detected for individual autoanitgen in the multiplex system. (A) Comparison of the p53 coupled beads in a singleplex (individual bead) and multiplex (multiple beads) system. The MFI value was detected by a series dilution of p53 antibody. (B) No cross-reactivity between different biomarkers in the multiplex system. A multiplex system containing five microspheres coupled with NY-ESO-1, p53, p62, PRDX and HaloTag separately was incubated with p53 antibody, and only the p53 microspheres reacted with anti-p53 antibody, while other microspheres detected the background MFI values. (C) The signal correlation of the p53 microspheres in the singleplex and multiplex system. (D) Same as (C) but using p62 coupled microspheres.

We next developed the multiplexed Luminex system. Four protein-microsphere complexes (NY-ESO-1, p53, p62, and PRDX) as well as a HaloTag conjugated bead as reference control were equally combined and distributed into a 96-well plate. The anti-p53 antibody was used to detect the p53 microsphere in this multiplex system. The data showed that the MFI signals were highly correlated with those of the individual bead system (single-plex) (**Fig. 4A**) demonstrating the multiplex system is valid for autoantibodies detection. In addition, except the p53 microsphere, all other microspheres including NY-ESO-1, p62, PRDX produced the background signals indicating there was no cross-reactivity between the beads in this multiplex system (**Fig. 4B**).

We next sought to test the correlation of both single- and multi-plex Luminex systems by comparing the autoantibody signals of randomly selected serum samples detected by both systems separately. As showed in **Fig. 4C** and **Fig. 4D**, the correlation coefficient (Pearson r) was 0.84 for the p53 microsphere (*p*<0.01), while 0.81 for the p62 microsphere (*p*<0.01), The Pearson r was also above 0.80 for other protein-microsphere complexes (data not shown) indicating the high correlation between single- and multi-plex systems.

**Figure 5:**
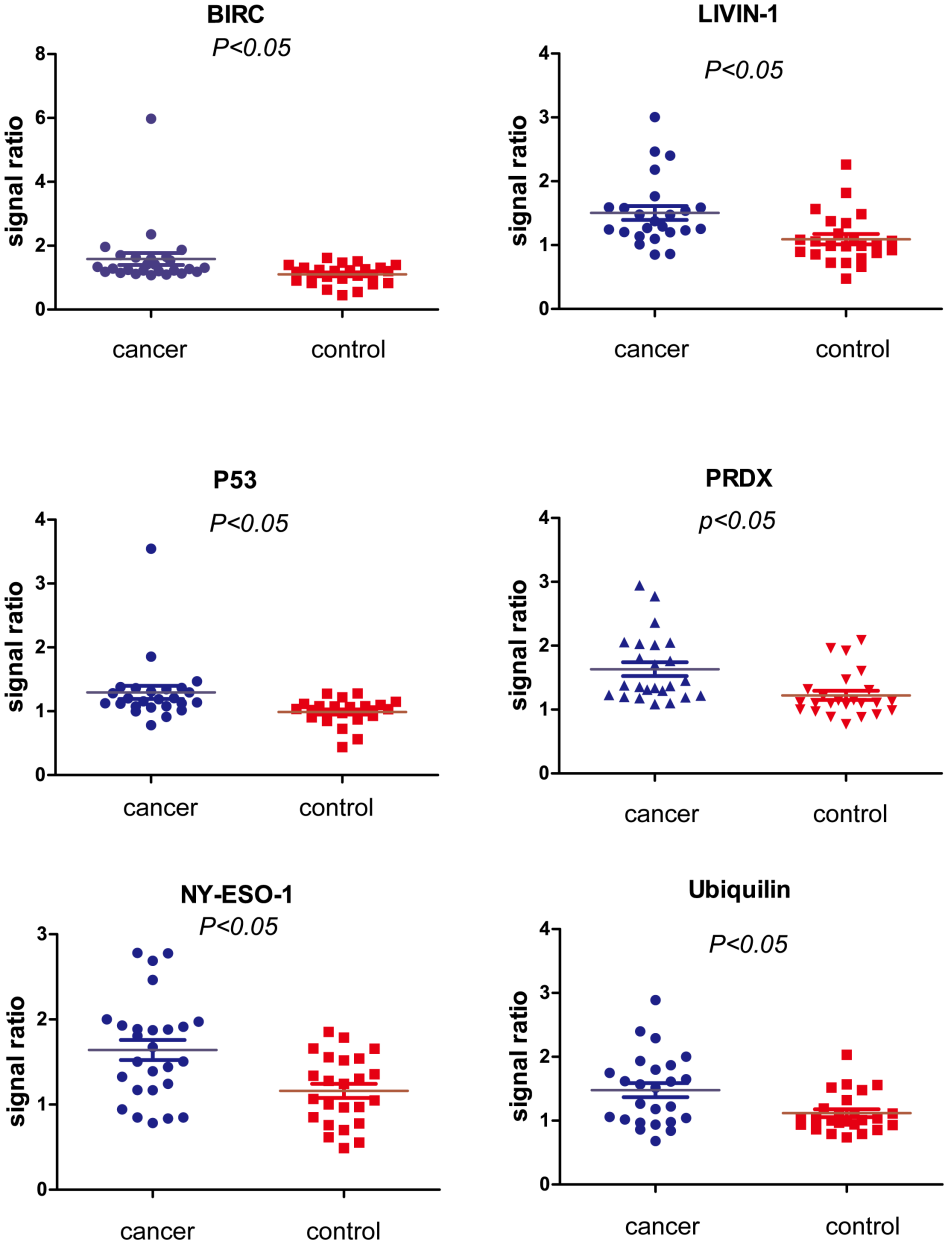
Scatter diagrams of the autoantibodies in 25 cancer sera and 25 controls detected by Luminex system using six TAAs (p53, NY-ESO-1, Livin-1, Ubquilin, BIRC and PRDX).

Based upon the multiplex Luminex system, we screened a panel of 14 candidate autoantigens to detect the circulating antibodies from independent 25 lung cancer and 25 healthy control randomly selected from the sample cohort in this study. Seven autoantigens, including p62, BIRC, Livin-1, p53, PRDX, NY-ESO-1 and Ubiquilin, produced the remarkably higher MFI signals in lung cancer patients compared to healthy controls (Mann-Whitney p value <0.05)(**Fig. 5**).

**Figure 6:**
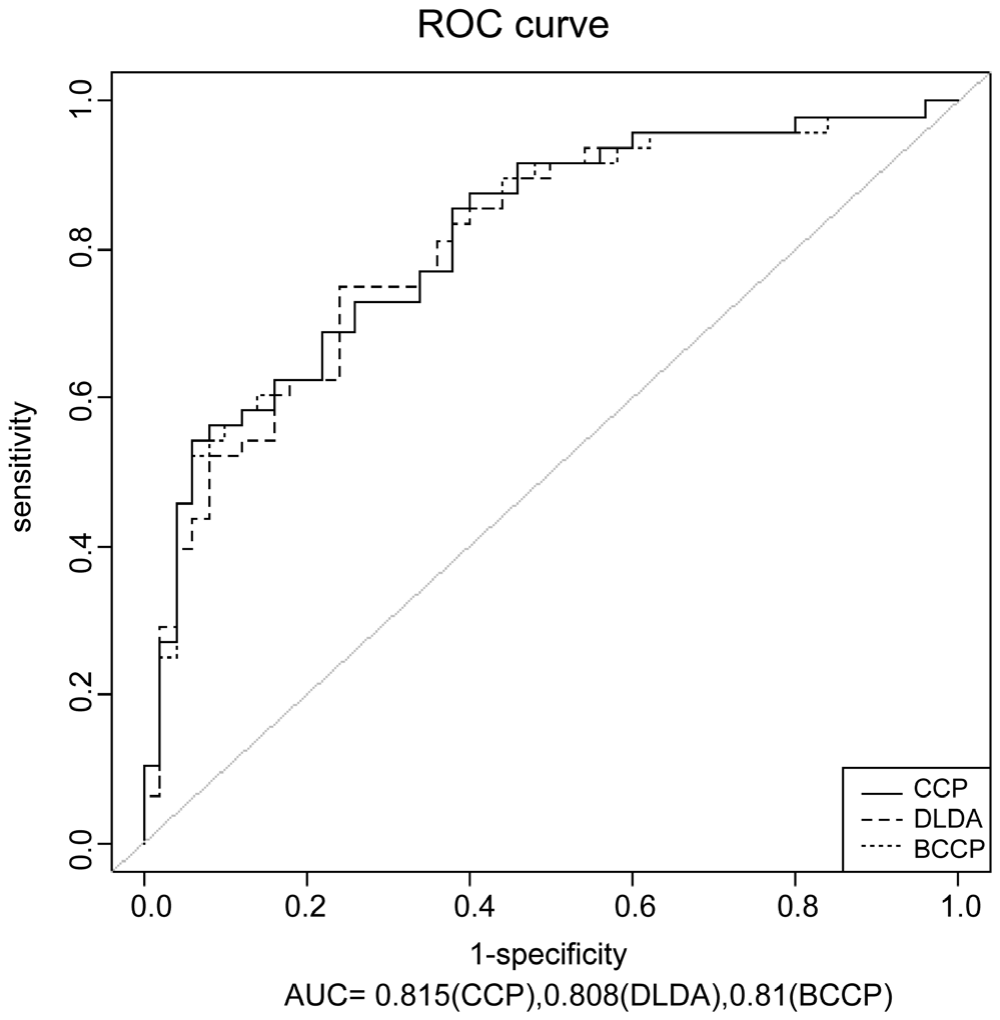
The ROC curves of the seven biomarkers calculated by three models: CCP, DLDA, BCCP.

We next determined whether the panel of 7 biomarkers could be used for the noninvasive detection of lung cancer patients. We screened independent 48 lung cancers and 50 control sera samples with various lung cancer types and stages by the Luminex multiplex system. The basic parameters of the 48 cancer patients and 50 controls are summarized in **Table 1**, including age, sex, smoking and drinking status. After separation into cancer and control classes, the accuracy of binary outcome prediction was estimated using different machine learning algorithms available at BRB Array Tools. Each sample’s value for each of those 7 biomarkers was multiplied by the corresponding coefficients derived from univariate logistic regressions on the training set with cancer/control as a binary response variable, and then the values were totaled. The created index scores were then assessed by the ROC curve, which provided a pure index of a test’s accuracy by plotting the sensitivity against 1– specificity for each result value of the test. Three prediction algorithms were used to generate the ROC, including compound covariate predictor (CCP), diagonal linear discriminant analysis (DLDA), and Bayesian compound covariate predictor (BCCP). Notably, the analysis yielded a very comparable ROC for all three algorithms with AUC of 0.82(CCP), 0.81(DLDA), 0.81(BCCP) respectively (**Fig. 6**), demonstrating the strong discriminative power of the 7 biomarkers. No correlation with age, gender, smoking or drinking status was observed for this biomarker panel.

**Table 1:** Basic parameters of the lung cancer patients and healthy controls.

	Cancer	Healthy
**Age(years)**		
Range	39–79	29–76
Mean±SD	59.7±8.7	58.4±12.9
≥50	42	35
≤50	6	15
**Gender**		
Male	34	36
Female	14	14
**Smoking Status**		
Non-smoking	24	
Smoking	24	
**Drinking Status**		
Non-drinking	34	
Drinking	14	

## Discussion

Detection of the circulating antibodies against tumor associated antigens is a promising approach for early diagnosis of cancer, and the technologies have been widely developed [14], [15]. To date, ELISA is the conventional method for detecting the autoantibody in serum, but its application on multiplexing autoantibody signature is limited. Recently, protein microarray and Luminex xMAP technologies were adopted for measuring the serological autoantibody panel [16], [17].

Luminex xMAP is a high throughout platform with superior sensitivity and specificity than ELISA [18], [19], [20]. In this study, we described a simple Luminex method based on HaloTag technology to detect the autoantibodies from patient sera. The HaloTag proteins were covalently bound to the chloroalkane linkers conjugated to the microspheres [21], [22]. Due to the specific covalent binding, the raw Halo-tagged fusion protein lysates are selectively immobilized to the Luminex beads and subsequently go through vigorous washing step, thus skipping the tedious protein purification procedure.

It is well established that multiplexing single biomarkers (i.e. autoantibody profiling) could significantly increase the sensitivity and specificity of cancer biomarkers in discriminating the cancer patients from healthy controls [23]. The aim of this study was to take advantage of Luminex technique to multiplex a panel of 14 tumor-associated autoantigens in detecting lung cancer patients. These autoantigens were selected based on their performance in distinguishing cancers from healthy controls described in the literature. For example, p53 autoantibody was detected in about 15% of cancer patients [24], [25]. Ubiquilin 1 was found to be a promising biomarker for lung cancer with an AUC of over 0.7 [26]. Livin-1 autoantibody was reported in 19 of 37 lung cancer patients (51.3%) [27]. In addition, 25 (47%) of 53 NSCLC patients were tested positive for autoantibodies against PRDX in the sera [28]. Autoantibody to NY-ESO-1, BIRC, and p62 were also detected in lung cancer patients with 20%, 19.5% and 18.8% sensitivity respectively [29], [30], [31].

Multiplex the tumor-associated antigens have been extensively explored by ELISA [32], [33], [34], [35], [36] or microarray [37]. This study is the first report by combining Luminex platform and HaloTag technology to detect humoral immune response in lung cancer patients. The panel of 7 biomarkers achieved over 80% accuracy in detecting lung cancer from healthy controls. These autoantibodies, however, have no association with tumor histologies, stages and types due to the limit of sample size. Therefore, follow-up study will be required in a large patient cohort with a mixture of tumor types and stages to validate the performance of the autoantibodies across tumor histologies and types, especially, the correlation between disease malignancy and the autoantibody titer. One can envision that this multiplex Luminex system as well as the panel of seven biomarkers could be used to screen the high-risk population with subsequent CT test based on the blood test result.

Exploiting the immune response to tumors provides a unique opportunity for developing new tools for the serological detection of cancer as well as a lead for therapy. A test based on the demonstration of autoantibodies to tumor antigens in sera of patients could be of great importance for early detection of cancer because of the prolonged time course of carcinogenesis and because a detectable level of antibodies against carcinogen stimulus could form well before the tumor phenotype arises. Like other cancers, lung cancer develops as the results of the derailment of heterogeneous and multiple regulatory processes. Therefore, it is not a single biomarker that needs to be elucidated. Multiplexed biomarker patterns have a significantly higher positive predictive value than single markers in discriminating diseased patients from non-cancer controls.

The autoantibody test holds great promise, but much more research with extensive samples is required to confirm the test’s reliability and to adapt the technique for mass screening. In addition, doctors will also need to learn if early diagnosis improves the outcome for patients with lung cancer, thus helping to produce antibodies for disease treatment. Therefore, bridging the gap between basic science and clinical practice represents the main goal in the near future to enable physicians to tailor risk adjusted screening and treatment strategies for current lung cancer patients.
